# A Comparative UHPLC-Q-Trap-MS/MS-Based Metabolomics Analysis to Distinguish *Foeniculum vulgare* Cultivars’ Antioxidant Extracts

**DOI:** 10.3390/molecules28020900

**Published:** 2023-01-16

**Authors:** Maria Assunta Crescenzi, Gilda D’Urso, Sonia Piacente, Paola Montoro

**Affiliations:** 1 Department of Pharmacy, University of the Study of Salerno, Via Giovanni Paolo II 132, I-84084 Fisciano, Italy; 2 Ph.D. Program in Drug Discovery & Development, Department of Pharmacy, University of the Study of Salerno, Via Giovanni Paolo II 132, I-84084 Fisciano, Italy

**Keywords:** metabolomics, antioxidant activity, multivariate statistical analysis

## Abstract

Among the environmental factors, seasonality is the one which most affects the metabolome of a plant. Depending on the harvest season, the plant may have a variable content of certain metabolites and thus may have different biological properties. *Foeniculum vulgare* is an annual plant whose cultivation creates large amounts of waste rich in bioactive compounds. The present investigation was performed with the aim of determining the amount of biologically active compounds in *F. vulgare* wastes obtained from varieties of different seasonality. Ten polyphenolic compounds were quantified in the little stems and leaves of Tiziano, Pegaso, and Preludio cultivars by ultra performance liquid chromatography (UPLC) hyphenated to QTRAP mass spectrometry by using the MRM (multiple reaction monitoring) method. The antioxidant activity of hydroalcoholic extracts was then evaluated using TEAC and DPPH spectrophotometric assays, followed by a multivariate statistical analysis to determine the correlation between metabolite expression and antioxidant activity. The Preludio variety, grown in summer, showed a higher content of bioactive compounds, which guarantees it a better antioxidant power; kaempferol 3-*O*-glucuronide, quercetin 3-*O*-glucuronide, and quercetin 3-*O*-glucoside are the polyphenolic compounds that could be mainly responsible for the antioxidant effect of fennel. The PLS chemometric model, which correlated quantitative data obtained by a sensitive and selective LC-ESI-QTrap-MS/MS analysis of antioxidant activity, resulted in a selective tool to detect the compounds responsible for the activity shown by the extracts in chemical tests.

## 1. Introduction

Fennel (*Foeniculum vulgare* Mill) is one of the most used plants in traditional medicine, showing a high number of pharmaceutical applications [1,2,3]. In vitro and in vivo studies [4] have shown several pharmacological activities of fennel including antimicrobial [5], antiviral [6], anti-inflammatory [7], apoptotic [8], cardiovascular [9], and antitumor [10,11].

Fennel, grown mainly in the Mediterranean area, is an annual species. The part of fennel that is sold and most frequently used in food market is the soft white bulb [12]. The remaining parts of the plant such as the leaves, and stems represent a waste product that is, however, rich in bioactive compounds [1,13,14].

Previous studies from our research group, through the metabolite profiling of fennel waste, highlighted that the little stems and leaves are the richest parts in terms of secondary metabolites containing important antioxidants such as phenolic acids, glycosylated flavonoids, and iridoids [15,16].

Antioxidants are molecules capable of reducing oxidation reactions in the human body and in food products [17,18]. Therefore, these compounds, in addition to being used as natural antioxidants in food preservation to extend food stability and storage life, can eliminate the free radicals generated by oxidation reactions in the human body that damage cells [19]. The abundance of free radicals is associated with the onset of chronic diseases; therefore, the daily intake of antioxidant compounds can play an important role in the prevention and/or treatment of these diseases [20,21].

It is widely known that environmental factors, among them seasonality, can qualitatively and quantitatively affect the occurrence of metabolites in plants [22]. Different varieties of the same plant could have an increase or a reduction of some compounds according to the season in which they grow. The variability in the content of secondary metabolites could also determine a variability in their biological activities, such as the antioxidant activity [23,24].

Quantitative plant metabolomics is a tool to improve the understanding of plant biochemistry and metabolism by providing accurate measurements of the concentrations of known metabolites occurring in various plant samples prior to statistical and bioinformatic analysis [25]. Targeted approaches focus on the analysis of specific groups of metabolites associated with specific metabolic pathways or classes of compounds. The complexity and dynamics of metabolism require multiple analytical platforms to cover the full spectrum of metabolites. Among them, mass spectrometry MS/MS provides a highly sensitive and selective quantitative analysis of metabolites and has the ability to identify metabolites. Meanwhile multifunctional mass analyzers operating in integrated, or hybrid configurations can further facilitate metabolite identification by obtaining high-resolution and accurate MS/MS spectra. Among them, triple quadrupole (QqQ) is considered a reference tool for the absolute quantification of small molecules due to its sensitivity and specificity using multiple reaction monitoring (MRM): the first quadrupole (Q1) of the MS selects and transmits the precursor ions to the second quadrupole (Q2) for further fragmentation [26]. Therefore, this research was aimed at quantifying some metabolites in three varieties of different seasonality of *F. vulgare*, Tiziano (winter cultivar), Pegaso (spring cultivar), and Preludio (summer cultivar). Hydro-alcoholic extracts of the little stems and leaves were analyzed through UPLC-ESI-QTRAP-MS/MS analysis in MRM.

The antioxidant activity of fennel waste was evaluated by two spectrophotometric assays, specifically DPPH (1,1-diphenyl-2-picrylhydrazyl radical) and TEAC (Trolox equivalent antioxidant capacity). The content of flavonoids was also assessed using an allumine chloride colorimetric assay. Using these assays, the antioxidant activities of the spring (Pegaso) and summer (Preludio) fennel variety extracts were compared with those of the winter variety (Tiziano), whose results were previously reported [15].

PLS (Partial least squares (PLS) analysis, a multivariate data analysis projection method, can classify samples according to their properties, identified as a Y variable in a correlation plot. By using PLS analysis, the metabolomic quantitative data were correlated to specific assay results. Thus, in the present work the most important metabolites involved in the antioxidant activity were identified.

The study confirmed that seasonality can alter the metabolome of plants; in fact, the Preludio variety, grown in the summer period, has a higher content of bioactive compounds such as quercetin 3-*O*-glucoside, quercetin 3-*O*-glucuronide, and feruloylquinic acid.

## 2. Results and Discussion

### 2.1. UPLC-ESI-QTRAP-MS/MS Quantitative Analysis

Ten known metabolites were quantified in fennel waste obtained from different cultivars by UPLC-ESI-QTRAP-MS/MS analysis. Data obtained from the Tiziano variety have already been published previously [16], they are reported for a comparison with the other two varieties of different seasonality.

**Figure 1:**
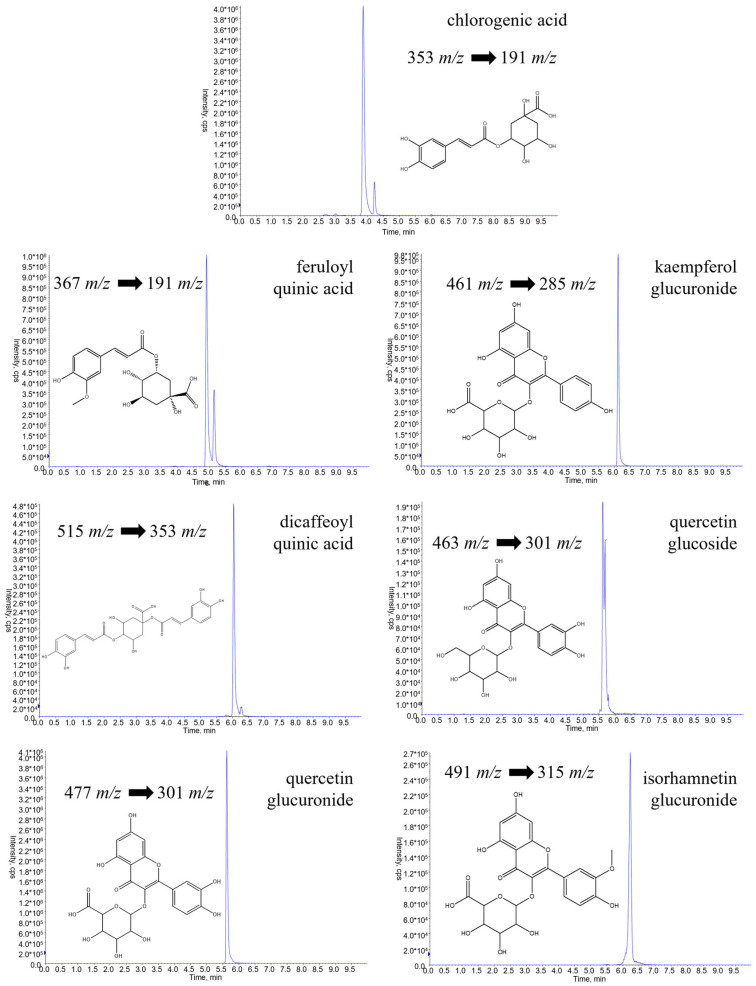
Q1/Q3 mass transitions for chlorogenic acid, quercetin glucoside, quercetin glucuronide, kaempferol glucuronide, isorhamnetin glucuronide, feruloylquinic acid, and dicaffeoylquinic acid obtained by UHPLC-ESI-QTRAP-MS/MS analysis and selected for MRM analysis of a standard mix.

The mass spectral parameters of each compound were optimized according to standard methods, as shown in Table 1, along with a precursor/production transition selected to apply an MRM method for revealing them (Figure 1).

**Figure 2:**
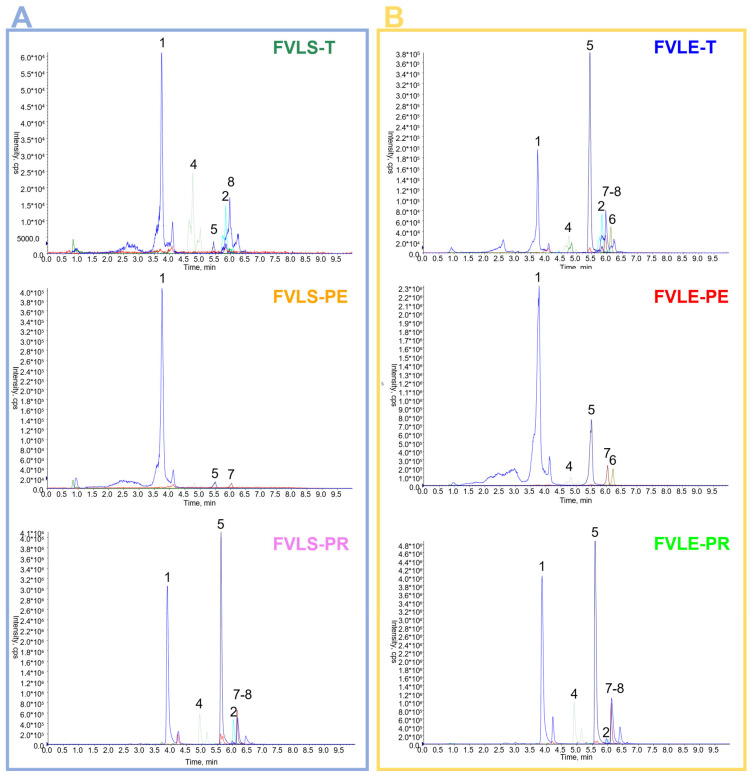
UHPLC-ESI-QTRAP-MS/MS profiles of hydroalcoholic extracts of the little stems (**A**) and leaves (**B**) of three varieties of *F. vulgare*. FVLS-T, *Foeniculum vulgare* little stem Tiziano variety; FVLS-PE, *Foeniculum vulgare* little stem Pegaso variety; FVLS-PR, *Foeniculum vulgare* little stem Preludio variety; FVLE-T, *Foeniculum vulgare* leaf Tiziano variety; FVLE-PE, *Foeniculum vulgare* leaf Pegaso variety; FVLE-PR, *Foeniculum vulgare* leaf Preludio variety.

For both the Tiziano and Pegaso varieties, the leaves showed a higher concentration of the quantified compounds (Table 2). On the contrary for the Preludio variety, the little stems were the richest source of bioactive compounds (Figure 2A).

Neochlorogenic acid (**1**) and quercetin 3-*O*-glucoside (**3**) were abundant in the leaf of the spring variety, Pegaso, with concentrations of 192.67 and 90.36 mg, respectively. Additionally, this variety showed the greatest concentration of isorhamnetin 3-*O*-glucuronide (**6**) (888.86 mg) (Figure 2B). Kaempferol 3-*O*-rutinoside (**9**) and kaempferol 3-*O*-glucoside (**10**) were detected only in the fennel waste from the Preludio variety. A higher concentration of bioactive compounds was found in the little stem of this variety of fennel. In fact, it was rich in glucoronate flavonoids such as quercetin 3-*O*-glucuronide (**5**) and kaempferol 3-*O*-glucuronide (**7**). These flavonoids were present at 1611.00 and 562.50 mg concentrations, respectively. In the little stem of Preludio fennel, grown during the summer, the highest concentrations of feruloylquinic acid (**4**) and quercetin 3-*O*-glucoside (**3**) were detected, with concentrations of 310.50 and 264.60 mg respectively in 100 g of dried plant material.

### 2.2. Method Validation

According to EMA guidelines, the UPLC-ESI-QTRAP-MS/MS method has been validated as previously described [16,27]. Each standard solution was analyzed in triplicate to assess a calibration curve by plotting the area of the external standard alongside each metabolite concentration. The linearity was evaluated considering the correlation coefficients of each calibration curve obtained for standard compounds. The correlation values obtained were in the range of 0.997–0.999.

A signal-to-noise ratio (S/N) of 3:1 and 10:1 was obtained by serially diluting the standard compounds under optimized conditions in order to determine, respectively, the limit of detection (LOD) and the limit of quantification (LOQ). Therefore, the developed method demonstrated good sensitivity with a LOD between 0.002 and 0.010 mg/L and a LOQ between 0.002 and 0.08 mg/L.

A sample was analyzed three times on the same day and three times over three consecutive days, in order to evaluate the precision of the method; the value was expressed as a percentage relative standard deviation (RSD). The RSD value for any analite was in the range of 2–4%. Using the optimized parameters, we performed recovery experiments to evaluate the extraction efficiency.

At three concentration levels (high, middle, and low), UPLC-ESI-QTRAP-MS/MS experiments were performed in triplicate at each concentration level for three standard solutions. The recovery and precision were appreciable, with recovery rates ranging from 95% to 105% on the same day.

### 2.3. Antioxidant Activity and Content of Flavonoid

Flavonoids are phenolic compounds which are very abundant in fennel waste. These compounds exert antioxidant, antimicrobial, photoreceptor, feeding, and light-screening functions in plants [28]. Research shows that flavonoids also have antiallergenic, antiviral, anti-inflammatory, antioxidant, and vasodilating properties in the human organism [29]. Due to the presence of glycosylated flavonoids in fennel waste, the flavonoid content and antioxidant activities of extracts were chemically tested using spectrophotometric methods (Table 3).

There was a higher flavonoid content in the leaves than in the little stems of all the varieties tested. In particular, the leaves of the summer variety, Preludio, presented the richest source of flavonoid compounds with a concentration of 1.497 mg. Although each extract showed good antioxidant activity, by comparing the results with those obtained for the two positive controls (quercetin 3-O-glucoside and vitamin C), the leaves in general, compared to the little stems, were shown to be more active. Specifically, according to the data from the quantitative analysis, the Pegaso and Preludio varieties exerted the highest antioxidant activity-reaching TEAC values (equivalent antioxidant capacity in TROLOX expressed in mg/mL) of 1.501 for FVLE-PR and 1.676 for FVLE-PE and, respectively, an IC_50_ for DPPH (expressed in mg/mL) of 0.010 for FVLE-PR and 0.634 for FVLE-PE.

### 2.4. Multivariate Data Analysis

Quantitative data obtained for biological triplicates of the various samples were used to apply a multivariate statistical analysis with a targeted approach.

A data matrix was created where the rows (variables) were the quantities of each metabolite analyzed by LC-ESI-QTRAP-MS/MS (Table 2) and the columns were the various samples, each in triplicate, and represented the observations. This matrix was the X-block for the PLS-based approach, which is a regression technique applied to examine the relationship between two blocks of data, called X- and Y-block. The Y-block is the antioxidant activity, expressed as a % of inhibition of TEAC or DPPH. A score scatter plot generated by PLS highlights the extracts with the greatest antioxidant activity, evaluating their proximity to the Y-block. Additionally, a loading scatter plot shows the variables closest to the Y-block, which correspond to the variables that had a greater impact on antioxidant activity.

**Figure 3:**
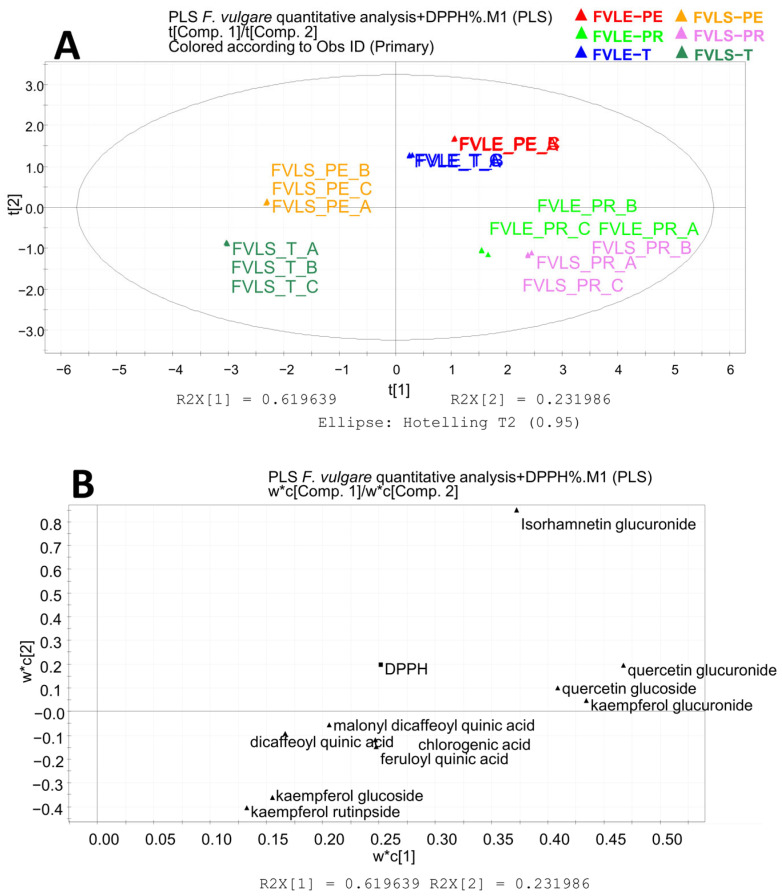
Partial least square analysis: quantitative results related to the antioxidant activity (DPPH) of the ethanolic extracts of *F. vulgare* waste. (**A**) Score scatter plot; (**B**) loading plot. FVLE-T, *Foeniculum vulgare* leaf Tiziano variety; FVLE-PE, *Foeniculum vulgare* leaf Pegaso variety; FVLE-PR, *Foeniculum vulgare* leaf Preludio variety.

**Figure 4:**
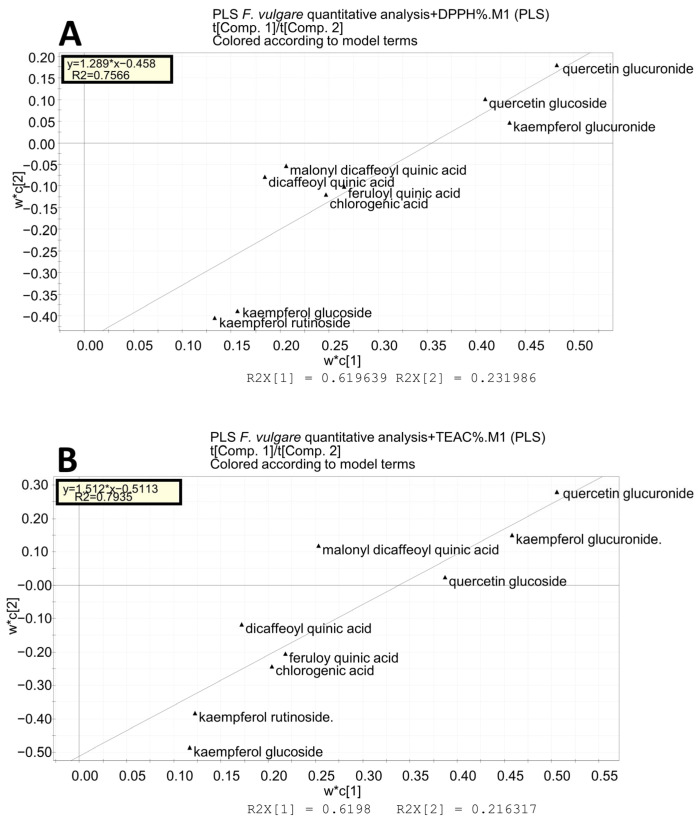
Partial least square analysis: loading scatter plots obtained from a targeted data analysis excluding the outlier isorhamnetin glucuronide. (**A**) Loading scatter plot with regression line with the Y-block as the DPPH value; (**B**) loading scatter plot with regression line with the Y-block as the TEAC value.

Figure 3 shows the score scatter plot of the PLS analysis for DPPH (panel A) and the loading scatter plot (panel B). The first component explains the 62% of variance, while the second explains the 23% of variance. The most active samples are located on the right side of the plot and are the leaves of all the varieties of fennel and the little stems of the Preludio summer variety. The loading scatter plot has an outlier, the isorhamnetin 3-O-glucuronide, which was eliminated to obtain a new loading scatter plot with the regression line (Figure 4A). The variables with the greatest antioxidant power are those positioned in the upper right part of the plot, the flavonoids quercetin 3-O-glucuronide, quercetin 3-O-glucoside, and kaempferol 3-O-glucuronide.

**Figure 5:**
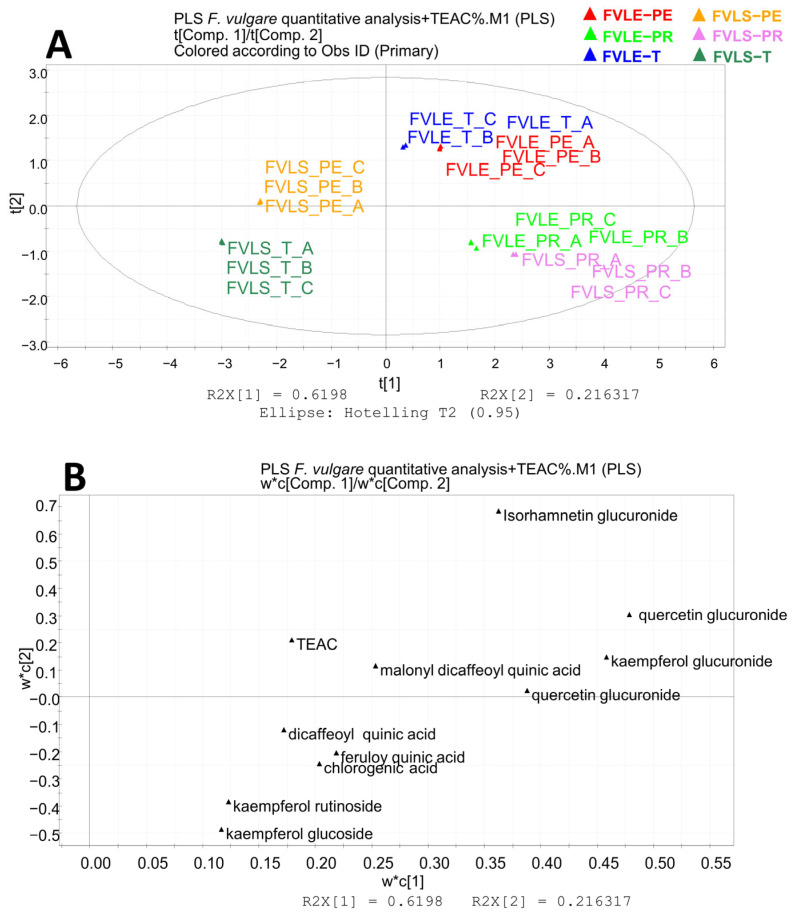
Partial least square analysis: quantitative results related to the antioxidant activity (TEAC) of the ethanolic extracts of *F. vulgare* waste. (**A**) Score scatter plot; (**B**) loading plot. FVLE-T, *Foeniculum vulgare* leaf Tiziano variety; FVLE-PE, *Foeniculum vulgare* leaf Pegaso variety; FVLE-PR, *Foeniculum vulgare* leaf Preludio variety.

A PLS analysis with TEAC inhibition as the Y-block is represented by Figure 5. The first component explains the 62% of variance, while the second explains the 22% of variance. The results of the score scatter plot are the same as obtained with the DPPH, thus confirming that Preludio leaves and little stems are the parts of fennel waste with the greatest antioxidant activity. Again, the isorhamnetin 3-O-glucuronide is an outlier and was eliminated. The variables that are responsible for the antioxidant power are confirmed as quercetin 3-O-glucuronide, quercetin 3-O-glucoside, kaempferol 3-O-glucuronide, and also malonyl dicaffeoylquinic acid (Figure 4B).

## 3. Materials and Methods

### 3.1. Raw Materials

A company specialized in the production and marketing of fennel, Paolillo (Eboli, Salerno, Italy), provided the by-products of *F. vulgare*. As part of the processing, the waste of fennel was recovered from the Tiziano variety harvested in December 2019 and from the Pegaso variety harvested in April 2021, both of which were grown in Campomarino in Molise, and from the Preludio variety harvested in July 2021, cultivated in Avezzano in Abruzzo. Superficial leaves and smaller stems of fennel were collected. The samples were classified into the following groups: FVLS-T (*F. vulgare* little stems of the Tiziano variety), FVLE-T (*F. vulgare* leaves of the Tiziano variety), FVLS-PE (*F. vulgare* little stems of the Pegaso variety), FVLE-PE (*F. vulgare* leaves of the Pegaso variety), FVLS-PR (*F. vulgare* little stems of the Preludio variety), and FVLE-PR (*F. vulgare* leaves of the Preludio variety).

### 3.2. Chemicals

The ethanol and water used for the extractions were bought from VWR (Milan, Italy). Acetonitrile (ACN), formic acid, water, and methanol of LC-MS grade were provided by Romil (Milan, Italy). The standards used for the optimization of the method—neochlorogenic acid, dicaffeoylquinic acid, quercetin 3-*O*-glucoside, feruloylquinic acid, quercetin 3-*O*-glucuronide, isorhamnetin 3-*O*-glucuronide, and kaempferol 3-*O*-glucuronide—were purchased from Sigma-Aldrich (Milan, Italy). Trolox (6-hydroxy-2, 5, 7, 8-tetramethylchroman-2-carboxylic acid), DPPH (2, 2-Diphenyl-1-picrylhydrazyl), K_2_S_2_O_8_ (potassium persulfate), PBS (Phosphate Buffered Saline), and ABTS (2, 2’-azino-bis-(3-ethylbenzothiazoline-6-sulfonic acid) were bought from Sigma-Aldrich (Milan, Italy).

### 3.3. Sample Preparation

Before freeze-drying, the fennel waste was separated into different parts and stored at −80 °C. The classes of samples were FVLS-T (*F*. *vulgare* little stems of the Tiziano variety), FVLE-T (*F. vulgare* leaves of the Tiziano variety), FVLS-PE (*F. vulgare* little stems of the Pegaso variety), FVLE-PE (*F. vulgare* leaves of the Pegaso variety), FVLS-PR (*F. vulgare* little stems of the Preludio variety), and FVLE-PE (*F. vulgare* leaves of the Preludio variety).

The freeze-dried plant materials were extracted by ultrasound assisted extraction as previously described [15]. For the sonication of the FVBU and FVST samples, 1 g of dried drugs were extracted with 20 mL ethanol/water (80:20) for 15 min in an ultrasonic bath. In contrast, the extraction of the FVLS and FVLE samples required 40 mL for 1 g of the matrix. The extraction was repeated three times and the extracts were filtered with filter paper 67 g/m^2^ (530-16100-Aptaca). For LC-MS analysis, the combined extracts, dried under a nitrogen stream, were dissolved in methanol with a final concentration of 1 mg/mL.

### 3.4. Quantitative Analysis

#### 3.4.1. ESI-QTRAP-MS and ESI-QTRAP-MS/MS Analyses

The standard samples were analyzed using an ABSciex 6500 QTRAP spectrometer (Foster City, CA, USA) using ESI-QTRAP-MS/MS with full-scan and collision-induced dissociation (CID) analyses.

Flow rates of 10 µL/min were used to infuse a standard solution of each metabolite (1 µg/mL in methanol) into the source to optimize the analytical parameters. The data were acquired using the negative ion MS and MS/MS mode.

#### 3.4.2. UPLC–ESI-QTRAP-MS/MS Analyses in MRM (Multiple Reaction Monitoring) Mode

The bioactive compounds of fennel waste were analyzed quantitatively using a Shimadzu Nexera LC system in line with a Sciex 6500 QTRAP MS equipped with an Omega C18 column (Phenomenex, Aschaffenburg, Germany) (100 × 2.1 mm i.d., 1.6 μm). The mobile phases used were water + 0.1% formic acid (A) and acetonitrile + 0.1% formic acid (B). The gradient used at a flow rate of 0.300 mL/min, using the following increasing linear gradient (*v*/*v*) of solvent B was: 0.1–2.13 min, from 5% to 15%; 2.13–6.40 min, from 15% to 35%; 6.40–8.53, from 35% to 80%, and then back to 5% for 1.53 min. The ion mode was negative, and 5 μL of each sample was used for injection. The 6500 QTRAP was set up for IonSpray operation, and compounds were detected using multiple reaction monitoring (MRM). The mass spectrometry source parameters were set as follows: curtain gas (CUR) = 35; collision gas (CAD) = medium; ion spray voltage (IS) = −4500; temperature (TEM) = 350; ion source gas 1 (GS1) = 25; ion source gas 2 (GS2) = 25. [16]. The transitions of each analyzed metabolite are listed in Table 1 with the following parameters: declustering potential (DP), entrance potential (EP), collision energy (CE), collision cell exit potential (CXP). The dwell time for each analyte was 20 ms. There were typically 14 points across all chromatographic peaks with a total cycle time of 0.3 s. Analyst software 1.6.2 was used for the data acquisition and processing (ABSciex, Foster City, CA, USA).

#### 3.4.3. Method Validation

The UPLC-ESI-QTRAP-MS/MS method was validated according to the European Medicines Agency guidelines (EMA quality guidelines ICH Q2) to validate the analytical methods following the procedure of the previous work [16,27].

### 3.5. DPPH Radical Scavenging Activity

The antiradical activity against 1,1-diphenyl-2-picrylhydrazyl radical (DPPH•) was evaluated in the stems and leaves of the three varieties of *F. vulgare*.

Extracts with antioxidant activity can reduce DPPH• to DPPH-H. As a result, there is a decrease in absorbance at 517 nm and it was measured on a UV-visible spectrophotometer (Spectrophotometer Multiskan Go, Thermo Scientific). The procedure of the assay was previously described [30]. The fennel extracts were tested with the following concentrations: 0.625–1.25–2.5 and 5 mg/mL, and the assay was performed in triplicate. The antioxidant power of vitamin C was evaluated as a positive control. The percentage of the radical inhibition of DPPH was calculated by the following Equation (1):

(1) $$\%\;Inhibition\;\;DPPH•=\left(1-\frac{A_{treated}-A_{blank}}{A_{control}}\right)\times 100\;$$

where *A_control_* is the average absorption of DPPH, while *A_treated_* is the average absorption of the extract with DPPH, and *A_blank_* is the average absorption of the extract solution only. The concentration of an extract that provides 50% DPPH inhibition (IC_50_) was calculated by plotting the inhibition (%) against each extract concentration.

### 3.6. Trolox Equivalent Antioxidant Capacity (TEAC) Assay

In line with previous works [15,31], the antioxidant capacity of the extracts was measured using the Trolox equivalent antioxidant capacity assay.

The TEAC value shows the ability of the antioxidant to scavenge the radical cation 2,2′-azinobis (3-ethylbenzothiazoline-6-sulfonate) ABTS·^+^ by spectrophotometric analysis. The ABTS·^+^ solution was prepared by mixing 7 mM ABTS in H_2_O with 2.45 mM potassium persulfate, and was stored in the dark at room temperature for 12 h. Subsequently, it was diluted with PBS (phosphate saline buffer, pH = 7.4), until an absorbance of 0.7 was reached at 734 nm and equilibrated at 30 °C.

The fennel extracts were diluted with methanol/water producing solutions at concentrations of 250, 500, 750, and 1000 mg/mL, respectively. A 96-well plate was used for the assay, containing 15 µL of each sample and 150 µL of ABTS. The absorbance was measured immediately at 734 nm. Triplicates of all the experiments were conducted.

As a function of the concentration of 6-hydroxy-2,5,7,8-tetramethylchroman-2-carboxylic acid (Trolox), the percentage decrease in absorbance was calculated for each concentration relative to a blank absorbance (methanol/water).

As a measure of antioxidant activity, the TEAC values were calculated from the concentration of a standard Trolox solution having the same antioxidant capacity as 1 mg/mL of the tested extract. As a reference compound, quercetin 3-*O*-glucoside was used.

### 3.7. Total Flavonoid Assay

An allumine chloride colorimetric assay was used to measure the total flavonoid content utilizing rutin as a standard according to the procedure previously described [15,32]. In a 10 mL volumetric flask, 1 mL (1 mg/mL) of each sample was mixed with 4 mL of water. The flask was then filled with 0.3 mL of 5% NaNO_2_. After 5 min, 0.3 mL of 10% AlCl_3_ was added. After 6 min, 2 mL of 1 M NaOH was added, and finally the solution was made up to a volume of 10 mL with water. The absorbances of the samples and the blank were measured at 510 nm in a UV-Vis spectrophotometer. According to Equation (2), the flavonoid content in different extracts is rutin equivalent (RE):

(2) $$Flavonoid\;amount=\frac{A\;*\;m_{0}*10\;}{A_{0}*\;m}$$

The flavonoid content RE is expressed as mg/g plant extracts. In the equation, *A* is the average of the absorbance of the extract in three samples, *A_0_* is the average of the absorbance of the rutin standard solution in three samples, *m* is the weight of the analyzed plant extract in g, and *m_0_* is the weight of the rutin in the solution in g.

### 3.8. Multivariate Data Analysis

A multivariate statistical analysis was applied with a targeted approach to better understand the relationship between the amount of bioactive compounds most expressed in fennel extracts and their antioxidant activity [33]. A data matrix was created in which the rows represented the different samples analyzed and the columns represented the content of the different metabolites quantified by LC-ESI-QTrap-MS/MS analysis. Another column was created, used as component Y, in which the antioxidant activity, expressed as a percentage of inhibition, was added for each sample calculated by either DDPH assay or TEAC assay.

PLS is a regression technique used to relate two sets of data [34]. The dataset was processed using SIMCAP+ 12.0 software (Umetrix AB, Umea, Sweden) for PLS, an approach for modeling the covariance structures between two spaces to find the fundamental relationships between two matrices; X is the amount of bioactive compounds in fennel extracts and Y is the antioxidant activity. Before performing the multivariate data analysis, Pareto scaling was applied to normalize the data. The PLS method was validated thought Hotelling’s permutation test and the T2 test. The significance of the mode was confirmed by the Q2 values of 0.8 and 0.4, respectively, in the models with DPPH and TEAC as Y-blocks.

## 4. Conclusions

The differences in the quantitative content of flavonoids in fennel varieties characterized by different seasonality showed an interesting variability in terms of flavonoids and phenylpropanoids, for example for neochlorogenic acid, quercetin 3-*O*-glucuronide, and dicaffeoylquinic acid malonyl.

The chemometric model obtained by the correlation of quantitative data, obtained by sensitive and selective LC-ESI-QTrap-MS/MS analysis of antioxidant activities with a PLS regression model proved to be a selective tool to detect the compounds involved in the antioxidant activities demonstrated by the extracts in chemical tests. The study allowed the identification of quercetin-3-*O*-glucuronide and kaempferol-3-*O*-glucuronide as the compounds that better contribute to the evaluated activity, independent of the extract under investigation. Similar correlation methods, based on multivariate data analysis and LC-MS, can be applied to better define the metabolites involved in the bioactivity of extracts of other plants with an interesting nutraceutical potential.

## Figures and Tables

**Table 1:** Mass spectral parameters and precursor/product MRM transitions of standard compounds measured using a UHPLC system interfaced with an ABSciex Q-Trap 6500 instrument in MRM mode (UPLC-ESI-QTRAP-MS/MS).

Compounds		DP	EP	CE	CXP	PI	DI
1	neochlorogenic acid	−60	−4	−24	−17	353	191
2	dicaffeoylquinic acid	−61	−4	−24	−38	515	353
3	quercetin 3-*O*-glucoside	−138	−9	−28	−32	463	301
4	feruloylquinic acid	−89	−4	−33	−21	367	191
5	quercetin 3-*O*-glucuronide	−52	−8	−35	−30	477	301
6	isorhamnetin 3-*O*-glucuronide	−52	−8	−35	−30	491	315
7	kaempferol 3-*O*-glucuronide	−59	−8	−35	−30	461	285
8	dicaffeoylquinic acid malonyl *	−61	−4	−24	−38	601	395
9	kaempferol 3-*O*-rutinoside **	−81	−4	−35	−30	593	285
10	kaempferol 3-*O*-glucoside **	−81	−4	−25	−30	447	285

DP, declustering potential; EP, entrance potential; CE, collision energy; CXP, collision cell exit potential; PI, product ion; DI, daughter ion. * Compound quantified on the dicaffeoylquinic acid curve. ** Compounds quantified on the kaempferol 3-*O*-glucuronide curve.

**Table 2:** Quantitative results obtained from the analyses of selected phenolic compounds in the *F. vulgare* waste of different cultivars using UHPLC-ESI-QTRAP-MS/MS analyses in MRM mode.

Compounds		FVLS-T	FVLE-T	FVLS-PE	FVLE-PE	FVLS-PR	FVLE-PR
**1**	neochlorogenic acid	6.80 ± 0.06	17.10 ± 0.86	28.84 ± 0.65	192.67 ± 3.34	151.74 ± 2.50	82.12 ± 2.63
**2**	dicaffeoylquinic acid	11.15 ± 0.21	41.17 ± 0.00	0.82 ± 0.02	1.71 ± 0.11	89.37 ± 2.42	22.88 ± 3.52
**3**	quercetin 3-*O*-glucoside	3.16 ± 0.67	50.85 ± 0.00	3.24 ± 0.05	90.36 ± 0.59	264.60 ± 3.09	81.60 ± 6.25
**4**	feruloylquinic acid	42.47 ± 0.00	46.77 ± 1.20	9.58 ± 0.36	103.13 ± 2.09	310.50 ± 5.91	185.60 ± 1.28
**5**	quercetin 3-*O*-glucuronide	1.14 ± 0.11	163.76 ± 2.61	11.14 ± 0.23	427.88 ± 8.36	1611.00 ± 16.11	872.00 ± 4.67
**6**	isorhamnetin 3-*O*-glucuronide	0.89 ± 0.13	257.90 ± 4.23	9.98 ± 1.02	888.86 ± 10.76	12.29 ± 1.07	5.26 ± 0.70
**7**	kaempferol 3-*O*-glucuronide	0.14 ± 0.00	37.84 ± 1.28	19.42 ± 0.26	238.76 ± 6.69	562.50 ± 10.73	366.40 ± 5.40
**8**	dicaffeoylquinic acid malonyl *	19.98 ± 1.01	48.18 ± 4.65	nd	0.61 ± 0.00	147.97 ± 5.46	107.72 ± 2.49
**9**	kaempferol 3-*O*-rutinoside **	nd	nd	nd	nd	16.92 ± 0.87	8.09 ± 1.13
**10**	kaempferol 3-*O*-glucoside **	nd	nd	nd	nd	15.24 ± 1.69	6.64 ± 0.75

Mean in mg/100 g dried weight with standard deviation. FVLS-T, *Foeniculum vulgare* little stem Tiziano variety; FVLE-T, *Foeniculum vulgare* leaf Tiziano variety; FVLS-PE, *Foeniculum vulgare* little stem Pegaso variety; FVLE-PE, *Foeniculum vulgare* leaf Pegaso variety; FVLS-PR, *Foeniculum vulgare* little stem Preludio variety; FVLE-PR, *Foeniculum vulgare* leaf Preludio variety. * Compound quantified on the dicaffeoylquinic acid curve. ** Compounds quantified on the kaempferol 3-*O*-glucuronide curve. nd, not detected.

**Table 3:** Antioxidant activity of extracts of *F. vulgare* evaluated by TEAC and DPPH, Radical scavenging activity assays, and the total amount of plant flavonoids of fennel.

*F. vulgare* Extracts	TEAC [mg/mL ± SD ^a^] ^b^	DPPH [IC_50_ (mg/mL) ± SD ^a^]	Total Flavonoids [mg/g Plant Extract (in RE) ± SD ^a^] ^c^
FVLS-T	0.375 ± 0.006	5.782 ± 0.001	ND
FVLE-T	0.823 ± 0.008	0.342 ± 0.002	0.206 ± 0.006
FVLS-PE	0.448 ± 0.006	3.280 ± 0.002	0.083 ± 0.001
FVLE-PE	1.676 ± 0.010	0.634 ± 0.006	1.331 ± 0.002
FVLS-PR	0.509 ± 0.004	1.109 ± 0.002	0.309 ± 0.001
FVLE-PR	1.501 ± 0.009	0.010 ± 0.002	1.497 ± 0.002
quercetin 3-*O*-glucoside	1.813 ± 0.007	/	/
vitamin C	/	0.270 ± 0.006	/

SD ^a^, standard deviation of three independent experiments; ^b^, antioxidant activity determined by TEAC assay, and expressed as the antioxidant capacity equivalent in TROLOX in mg/mL; ^c^, flavonoid content evaluated by aluminum chloride assay and expressed as mg of rutin equivalents (RE) in grams of extract.

## Data Availability

The data presented in this study is contained within the article.

## Sample Availability

Samples of the compounds are available from the Pharmaceutical Biology Laboratory, Department of Pharmacy, University of Salerno, Via Giovanni Paolo II 132, I-84084 Fisciano, Italy.
